# The relationship between subjective compliance with isolation precautions and moral sensitivity in novice nurses: cross-sectional study

**DOI:** 10.1186/s12912-024-01735-0

**Published:** 2024-01-25

**Authors:** Hanife Tiryaki Şen, Şehrinaz Polat, Leyla Afşar Doğrusöz

**Affiliations:** 1 Training Nurse, Istanbul Health Directorate, Fatih, Istanbul, Türkiye Turkey; 2 Faculty of Nursing, Istanbul University, Fatih, İstanbul, Turkey; 3 Istanbul Faculty of Medicine, Istanbul University, Quality Coordinator, Istanbul, Türkiye Turkey

**Keywords:** Morals, Healthcare associated infection, Isolation, Nurses, Cross-sectional study

## Abstract

**Background:**

Increasing compliance with isolation precautions is important in reducing hospital-acquired infections and their consequences. It is not possible to achieve and maintain nurse compliance through supervision, control, pressure, or training. Therefore, nurses must personally demand compliance with isolation precautions. This study aimed to determine the relationship between compliance with isolation precautions and the moral sensitivity levels of nurses who have just started working.

**Methods:**

This study employed a descriptive and cross-sectional research design. The study population consisted of 456 new nurses recruited during the pandemic. All of the new nurses who volunteered to participate in the study and completed the questionnaires completely were included in the study and 398 nurses constituted the sample of the study. Data were collected from 398 out of 456 novice nurses who agreed to participate in the study. The population coverage rate was 87.28%. Participants were selected using convenience sampling method. The data collection tools included an Introductory Information Form, The Isolation Compliance Precautions Scale (TIPCS), and the Moral Sensitivity Questionnaire (MSQ). Descriptive statistics, correlation analyses, and regression models were used to analyze data.

**Findings:**

The mean score of the MSQ was high (mean ± SD = 90.49 ± 25.94; median (P25-P75) = 84 (range, 74–97), and the score for TIPCS was high (mean ± SD = 79.29 ± 7.68; median (P25-P75) = 82 (range, 76–85)). No correlation was found between MSQ and TIPCS (*p* > 0.05). According to the regression analysis, although the MSQ did not correlate with TIPCS, compliance with isolation measures was found to increase as the conflict subscale of moral sensitivity increased (β= -0.36, CI= -0.55 to -0.16; *p* < 0.001). Choosing the profession willingly had a positive correlation with compliance with isolation measures (β = 5.3, CI = 2.4 to 8.3; *p* < 0.001). In addition, starting the profession at an early age had a positive correlation with compliance with isolation measures (β= -0.49, CI= -0.8 to -0.17; *p* = 0.002).

**Conclusion:**

The conflict sub-dimension of moral sensitivity is an important factor in nurses’ compliance with isolation measures, and conducting necessary training and other studies to improve nurses’ moral sensitivity may increase their compliance with isolation measures.

## Introduction

Nurses constitute the largest group of healthcare providers in the world and their impact on the quality of healthcare is extremely important. However, nursing is a profession that requires moral effort [1]. Increased compliance with isolation measures contributes to the reduction of hospital-acquired infections (HAIs) and their consequences (e.g., costs, functional impairment, emotional distress, reduced quality of life, and death) [2–4]. The importance of compliance with isolation measures has once again become strikingly critical with the severe consequences of the COVID-19 pandemic. It is critical for nurses who spend time with patients to establish close relations with them during their care, and to take responsibility for patient care practices to follow isolation precautions to deliver safe and quality health service [5].

Dobrina [6] reported a statistically significant improvement in the rate of compliance with standardized isolation precautions between assessments before and during COVID-19 [6]. In the literature, studies have reported that the perception of the COVID-19 pandemic [7–8] and the fear of COVID-19 [8–9] increased healthcare workers’ compliance with isolation. On the other hand, in some studies conducted during the COVID-19 pandemic, compliance with isolation precautions was reported to be high [7, 10–11], moderate [12], and low [7, 13–14]. Some studies that have looked at both knowledge and compliance with isolation standards have found that knowledge is higher than compliance [15–16].

In their study, McCauley et al. [17] determined that five major themes, namely Nurses and Resource Organization, Workplace Environment, Nursing Care Context, Management and Interprofessional Relations, and Individual Nurse Factors, had an effect on both missed nursing care and noncompliance in the field of infection prevention and control.

Evaluating the moral sensitivity of nurses is also possible within the context of individual nursing factors. Moral sensitivity is associated with anxieties and moral dilemmas caused by the conflict between patients’ desires and the obligations and duties of professionals [18]. Moral sensitivity is the source of nursing ethics as well as a prerequisite for nurses’ service behavior [19] and has a positive effect on nursing behavior [20]. Ohnishi et al. [21] stressed that nurses with high moral sensitivity could perceive and define moral problems. The literature shows that care positively affects both patients and nurses. It is emphasized that the duration of hospital stays is reduced through the continuity of patient care and improved preventive care [20]. In a study conducted with nurses, it was reported that there was a positive correlation between moral sensitivity and caring behavior [22]. A study on intensive care nurses revealed a positive, significant, yet very weak correlation between overall moral sensitivity and the holistic approach, which is one of the subscales of moral sensitivity, and the level of principled thinking exhibited by intensive care nurses [23]. It is necessary to implement all methods that can increase compliance with isolation precautions and reduce hospital infection rates, and therefore the costs associated with the patients. To the best of our knowledge, although there are studies in the field of nursing conducted for compliance with isolation precautions, no study has examined the relationship between moral sensitivity and compliance with isolation precautions. Therefore, this study was planned to investigate the relationship between moral sensitivity and compliance with isolation precautions.

This study was conducted to determine the relationship between compliance with isolation measures and the level of moral sensitivity of nurses who were in the first 3 years of their nursing career and in the first month of their employment at the hospital where the study was conducted. In accordance with this purpose, answers to the following research questions were sought:

Q1: Is there a relationship between new nurses’ compliance with isolation measures and their level of moral sensitivity?

## Method

### Study design and participants

The study was descriptive, cross-sectional, and correlational. It was conducted in a public university hospital in the Marmara region of Türkiye, which receives patients from all over the country. The hospital provides tertiary care in all specialisms, and 456 nurses were recruited due to the increasing demand for nurses during the COVID-19 pandemic (October-December, 2020). The study focused on these nurses recruited during the pandemic, and a total of 456 new nurses constituted the study population. The data were collected between March and April 2021. All 456 nurses were included in the sample to increase the sample size because the study was conducted in a single hospital. Four hundred seven nurses agreed to participate in the study. Nine questionnaires were not analyzed due to incomplete responses. The sample of the study therefore consisted of 398 nurses who completed the questionnaires completely. Participants were selected using convenience sampling. The population coverage rate was 87.28%.

### Data collection

The data were collected between March and April 2021 using an Introductory Information Form, the Moral Sensitivity Questionnaire (MSQ), and The Isolation Compliance Precautions Scale (TIPCS). The novice nurses were informed about the purpose of the study. Those who volunteered to participate in the study were provided with a questionnaire, and the researchers collected the completed questionnaires after a week. It took 5–10 min to complete the data collection tool.

### Measures

#### Introductory information form

The form included a total of eight questions (age, sex, marital status, educational level, age of beginning the profession, willingness to choose the nursing profession, the status of choosing this profession if they had the opportunity to choose another one, and status of having any health condition) to determine the socio-demographic characteristics of the participants.

**Moral Sensitivity Questionnaire (MSQ)**: The scale was developed by Lutzen [24]. A validity and reliability study for the adaptation of the MSQ to the Turkish population was conducted by Tosun in 2018 [25]. The MSQ is used to test the moral sensitivity of physicians and nurses. This seven-point (1: strongly agree, 7: strongly disagree) Likert scale consists of 30 items and six subscales. A low score indicates high moral sensitivity and a high score indicates low moral sensitivity. The total score on the scale ranges between 30 and 210. The MSQ consists of a total of six sub-dimensions (Autonomy, Benevolence, Holistic Approach, Conflict, Practice, Orientation). The autonomy subscale of the questionnaire (items 10, 12, 15, 16, 21, 24, and 27) reflects respect for the principle of autonomy and patient preferences. The benevolence subscale (items 2, 5, 8, and 25) reflects actions aimed at increasing the benefit to the sick individual. The holistic approach subscale (items 1, 6, 18, 29, and 30) reflects actions that both do not harm the patient and protect the patient’s integrity. The conflict subscale (items 9, 11, and 14) reflects an internal experience of ethical conflict. The practice subscale (items 4, 17, 20, and 28) reflects thinking about the ethical dimension in deciding and implementing actions. The orientation subscale (items 7, 13, 19, and 22) reflects healthcare professionals’ interest in their actions that will affect their relationship with the patient. Items 3, 23, and 26 were not included in any of the subscales. Tosun found that Cronbach’s alpha value was 0.84 [25]. In the present study, Cronbach’s alpha coefficient was found as 0.886.

**The Isolation Compliance Precautions Scale (TIPCS)**: The development, validity, and reliability study of the scale was conducted by Tayran and Ulupınar in a sample of nurses, and Cronbach’s alpha value was found as 0.85 [26]. The scale is of the five-point Likert type and has 18 items that determine the compliance of nurses and physicians with isolation precautions. The lowest possible score to be obtained on the scale is 18, and the highest possible score is 90. A high score indicates a high level of compliance with isolation precautions. Items 18, 22, 24, and 34 are negative and are rated as follows: 1 = 5, 2 = 4, 3 = 3, 4 = 2, and 5 = 1. Cronbach’s alpha coefficient of the scale in this study was found as 0.844.

### Ethical considerations

The Ethics Committee of Istanbul University Istanbul Medical Faculty granted ethical approval to conduct the study (Date: 27/11/2020, Number: 246,159). The necessary information about the objective, scope, and ethical considerations of the study was provided on the first page of the data collection tool, and the consent of the participants was obtained. Permission from the author who adapted the MSQ into Turkish, and the author who developed TIPCS, was obtained. Those who agreed to participate in the study were not compensated, and there was no hierarchical relationship between these individuals and the researchers.

### Data analysis

The Statistical Package for the Social Sciences (SPSS) version 26 (IBM Corp., Armonk, NY, USA) was used for statistical analyses. Whether the scores obtained from each continuous variable were normally distributed was analyzed using descriptive, graphical, and statistical methods. The Kolmogorov-Smirnov test was used to test the normality of the scores obtained from a continuous variable in statistical methods. The reliability of the scales was evaluated using Cronbach’s alpha coefficient. Categorical variables are presented as frequency (n, %), and continuous variables are presented as mean ± standard deviation (SD), median, and quartiles (Q1 and Q3). Comparisons between two groups in continuous variables were performed using the Mann-Whitney U test. Comparisons of more than two groups were performed using the Kruskal-Wallis H test. Dunn’s multiple comparison test was used to determine which groups caused the difference in more than two group comparisons. The level of correlation between two continuous variables was analyzed using Spearman’s correlation test. A multivariate generalized linear model (GLM) was used to determine the independent variables associated with the dependent variables. Results were evaluated at 95% confidence intervals and significance was set at *p* < 0.05.

## Results

### Descriptive characteristics

A total of 398 nurses, 287 (72%) females and 111 (28%) males, with an average age of 24.24 ± 2.97 (range, 19–42) years, were included in the study. Of the nurses, 341 (86%) were single, 39 (10%) had a diagnosed health problem, 118 (30%) were high school graduates, and 301 (76%) started working at the age of 21 years or later. The findings revealed that 218 (55%) had one year of professional experience, 370 (93%) chose nursing willingly, and 314 (79%) would choose nursing again if they had a second chance (Table 1).

**Table 1:** Nurses’ personal and professional characteristics

Demographic variable (*n* = 398)	n	%
**Sex**		
Female	287	72.1
Male	111	27.9
**Marital status**		
Married	57	14.3
Single	341	85.7
**Age**		
19–22 years	118	29.6
23–25 years	179	45.0
26 years and older	101	25.4
**Education level**		
High School	118	29.6
Associate degree	179	45.0
Bachelor degree	101	25.4
**Age of beginning the profession**		
19–20 years	97	24.4
21–23 years	227	57.0
24 years and older	74	18.6
**Professional experience**		
1 year	218	54.8
2 years	69	17.3
≥ 3 years	111	27.9
**Choosing the profession**		
Willingly	370	93.0
Unwillingly	28	7.0
**Choosing the profession again**		
Yes	314	78.9
No	84	21.1
**Having any health condition**		
Yes	39	9.8
No	359	90.2

### Correlation between continuous variables

No statistically significant correlation was found between the total moral sensitivity level of the nurses and the level of compliance with patient isolation measures (*p* > 0.05); however, as the level of moral sensitivity related to autonomy (*r*= -0.116; *p* = 0.020), conflict (*r*= -0.230; *p* < 0.001), and orientation (*r*= -0.175; *p* < 0.001) increased (as the MSQ score decreased), the level of compliance with patient isolation measures increased statistically significantly. The level of correlation between the total and subscale scores of the nurses’ MSQ is given in detail in Table 2.

**Table 2:** Descriptive information about the variables

Variables	Mean ± SD	Median(P_25_-P_75_)	1	2	3	4	5	6	7
(1) Moral sensitivity	90.49 ± 25.94	84 (74–97)	N/A						
(2) Autonomy	19.19 ± 8.21	17 (13–23)	0.855^**^						
(3) Benevolence	12.89 ± 4.69	13 (10–16)	0.634^**^	0.454^**^					
(4) Holistic approach	13.17 ± 6.37	11 (9–15)	0.758^**^	0.637^**^	0.477^**^				
(5) Conflict	10.38 ± 3.89	10 (8–13)	0.190^**^	0.076	-0.053	0.105^*^			
(6) Practice	13.62 ± 4.30	13 (10–17)	0.670^**^	0.536^**^	0.371^**^	0.387^**^	-0.102^*^		
(7) Orientation	9.26 ± 5.83	7 (5–11)	0.768^**^	0.712^**^	0.439^**^	0.625^**^	0.198^**^	0.361^**^	
(8) Compliance with isolation precautions	79.29 ± 7.68	82 (76–85)	-0.094	-0.116^*^	-0.044	-0.084	-0.230^**^	0.066	-0.175^**^

**p < 0.01, *p < 0.05, **SD**: Standard deviation, Spearman correlation test, **N/A**: Not available

*Note: Negative relationships that are statistically significant due to the way the scales are calculated indicate the presence of a clinically positive relationship*

### Nurses’ level of moral sensitivity, level of compliance with isolation measures, and related factors

#### Univariate analysis results

The moral sensitivity level of nurses was evaluated using the MSQ. The mean total score was 90.49 ± 25.94; 23 (6%) of the nurses had a low level of moral sensitivity, 121 (30%) had a medium level, and 254 (64%) had a high level. When the level of moral sensitivity of the nurses was analyzed according to their descriptive characteristics, it was found that the level of moral sensitivity was lower in nurses who were female (Z= -2.839; *p* = 0.005) and had a health problem (Z= -2.422; *p* = 0.015) (Tables 2 and 3).

**Table 3:** Nurses’ level of moral sensitivity and adherence to isolation precautions by demographic characteristics

	MSQ			Compliance with Isolation Precautions		
Demographic variable (*n* = 398)	Median (P_25_-P_75_)	Test value	P-value	Median (P_25_-P_75_)	Test value	P-value
**Sex**		**-2.839** ^a^	0.005*		**-2.247** ^a^	0.025*
Female	86 (75–101)			82 (76–85)		
Male	82 (70–90)			80 (75–84)		
**Marital status**		-1.354^a^	0.176		-1.321^a^	0.187
Married	85 (78–99)			82 (79–86)		
Single	84 (73–97)			82 (76–85)		
**Age**		1.302^b^	0.522		1.687^b^	0.430
19–22 years	83 (73–95)			82 (78–85)		
23–25 years	85 (74–100)			82 (75–85)		
26 years and older	85 (74–99)			81 (75–85)		
**Education level**		4.283^b^	0.118		3.586^b^	0.166
High School	83 (72–95)			82 (78–85)		
Associate degree	79 (67–96)			80 (75–85)		
Bachelor degree	85 (75–101)			82 (74–85)		
**Age of beginning the profession**		5.277^b^	0.071		**7.936** ^**b**^	0.019*
19–20 years^1^	82 (70–97)			82 (79–86)	*dif = 1 > 2,3*	
21–23 years^2^	85 (76–99)			81 (76–84)		
24 years and older^3^	84(70–93)			81 (73–84)		
**Professional experience**		1.820^b^	0.403		**7.342** ^**b**^	0.025*
1 year^1^	85 (74–98)			80 (76–84)	*dif = 1 < 3*	
2 year^2^	80 (72–93)			82 (77–85)		
≥ 3 year^3^	85 (74–98)			82 (78–86)		
**Choosing the profession**		-0.057^a^	0.954		-1.669^a^	0.095
Willing	85 (74–97)			82 (76–85)		
Unwilling	82 (71–117)			79 (67–86)		
**Choosing the profession again**		-1.133^a^	0.257		-1.790^a^	0.073
Yes	84 (74–97)			82 (77–85)		
No	85 (75–109)			81 (73–84)		
**Having any health condition**		**-2.422** ^**a**^	0.015*		-1.036^a^	0.300
Yes	92 (80–121)			82 (77–85)		
No	84 (73–97)			82 (76–85)		

*p **< 0.05, P**_**25**_:25th percentile, **P**_**75**_:75th percentile, **a**:Mann-Whitney U test, **b**: Kruskal-Wallis H test

The mean level of nurses’ compliance with isolation measures was calculated as 79.29 ± 7.68 points. When the level of compliance with isolation precautions was analyzed according to the descriptive characteristics of the nurses, it was found that the level of compliance with isolation precautions was statistically significantly higher in female nurses (Z= -2.247; *p* = 0.025), those who started to work at an early age (< 20 years) (K-Wχ^2^ = 7.936; *p* = 0.019), and whose professional experience was 3 years or more (K-Wχ^2^ = 7.342; *p* = 0.025) (Tables 2 and 3).

#### Multivariate analysis results

Generalized linear regression model analysis was performed with a normal probability distribution and identity link function to determine the independent variables associated with the level of moral sensitivity of nurses by using the variables found to be statistically significant (*p* < 0.05) or close to significance (*p* < 0.1) in single-variable analysis results. When the correlation between the independent variables of the model and the dependent variable was examined, it was found that the only independent variable correlated with the level of moral sensitivity of nurses was sex, and the level of moral sensitivity of female nurses was found to be lower (β = 8.4, CI = 2.8 to 14.1; *p* = 0.004) (Table 4).

**Table 4:** Independent factors associated with nurses’ level of moral sensitivity *(generalized linear regression model analysis results)*

Variables	β	SE	95% CI (Lower-Upper)		Z	P-value
(Intercept)	85.225	4.706	76.001	94.448	18.111	< 0.001
Sex (Female)	8.404	2.881	2.758	14.051	2.917	0.004*
Age of beginning the profession	-0.833	1.967	-4.689	3.023	0.423	0.672
Having any health condition	8.397	4.323	-0.076	16.869	1.942	0.052

*p < 0.05; **Dependent variable**: Moral Sensitivity, **SE**: Standard error, **CI**: Confidence Interval, Generalized Linear Model, **Probability Distribution** = Normal, **Link Function** = Identity

Generalized linear regression model analysis was performed with a normal probability distribution and identity link function to determine the independent variables associated with the level of compliance of nurses with patient isolation measures by using the variables found to be statistically significant (*p* < 0.05) or close to significance (*p* < 0.1) in univariate analysis results. When the relationship between the independent variables of the model and the dependent variable was examined, it was found that the nurses’ willingly choosing the profession and doing it willingly (β = 5.3, CI = 2.4 to 8.3; *p* < 0.001), starting the profession at an early age (β=-0. 49, CI= -0.8 to -0.17; *p* = 0.002), and the level of conflict-related moral sensitivity (β= -0.36, CI= -0.55 to -0.16; *p* < 0.001) were found to be independent factors that increased compliance with patient isolation measures (Table 5).

**Table 5:** Independent factors associated with nurse compliance with isolation precautions *(generalized linear regression model analysis results)*

Variables	β	SE	95% CI (Lower-Upper)		Z	P-value
(Intercept)	86.596	4.080	78.599	94.593	21.223	< 0.001
Sex (Female)	1.442	0.820	-0.164	3.049	1.760	0.078
Choosing the profession (Willingly)	5.304	1.503	2.357	8.250	3.528	< 0.001*
Choosing the profession again	1.141	0.960	-0.740	3.021	1.189	0.235
Age of beginning the profession	-0.488	0.161	-0.804	-0.172	3.028	0.002*
Professional experience	0.242	0.184	-0.119	0.603	1.316	0.188
Autonomy	-0.033	0.088	-0.205	0.139	0.373	0.709
Conflict	-0.356	0.101	-0.554	-0.158	3.522	< 0.001*
Orientation	0.024	0.127	-0.226	0.273	0.187	0.852

*:*p* < 0,05; **Dependent variable**: Compliance with isolation precautions, **SE**: Standard error, **CI**: Confidence Interval, Generalized Linear Model, **Probability Distribution** = Normal, **Link Function** = Identity

## Discussion

The findings showed that moral sensitivity increased compliance with isolation precautions. In addition, the subscales of moral sensitivity, holistic approach, conflict, and practice also affected compliance with isolation measures.

In this study, the moral sensitivity score average of the nurses was found to be moderate. In some studies, it has been reported that moral sensitivity is at a high level [21, 23, 27, 28], whereas in other studies, nurses had a moderate level of moral sensitivity [22, 29].

The mean of compliance with isolation precautions was found to be at a good level. The findings of the present study are similar to some studies conducted during the COVID-19 pandemic in Türkiye [7, 10, 30–31]. However, there are also studies in the literature showing low compliance with isolation measures during COVID-19 [13, 32]. In a study conducted by Mehravar et al. [12] and Al-Faouri et al. [16] in Northern Jordan, it was reported that all nurses showed moderate compliance with standard precautions.

Although we found that those who started working before the age of 20 years had higher compliance with isolation measures, Dobrina reported statistically better compliance during COVID-19 in most age groups except for nurses aged ≤ 25 years [6]. There are also studies in the literature suggesting that compliance with isolation measures does not differ according to age [31, 33]. In a study by Küçük and Yarar, a significant correlation was found between age (25–30 years) and training on isolation methods [34].

In the present study, female nurses were found to comply with isolation measures more compared with male nurses. Similarly, some studies reported that female nurses were more compliant with isolation measures than male nurses [35, 36]. In the literature, studies show that compliance with isolation measures does not differ according to sex [31–32], but some studies indicate a difference [6–34]. In addition, contrary to the findings of the present study, there are also studies in the literature reporting that compliance with isolation measures is significantly higher in males than in females [37].

We found that the level of compliance with isolation measures was statistically significantly higher in nurses with a professional experience of 3 years or more. In the study of Arlı et al., it was reported that compliance with isolation increased as the duration of professional experience increased [36]. In the literature, there are also studies showing that compliance with isolation precautions does not differ according to the department of employment [31–35, 37].

No correlation was found between the total scale scores of moral sensitivity and the scale scores of compliance with isolation in the present study. Han et al. [32] determined that although compliance with standard precautions was significantly positively correlated with moral sensitivity and environmental safety in correlation analyses, the effect of moral sensitivity on compliance was not found in regression analyses. In their study, Amiri et al. [37] found no significant correlation between the quality of patient care and the moral sensitivity of nurses. In a study conducted with nurses, it was observed that as the moral sensitivity level of nurses increased, they displayed better care behavior [22]. In a study conducted with Chinese nurses caring for patients with COVID-19, it was reported that nurses’ positive perceptions of the ethical climate were both directly correlated with a higher level of self-assessed quality of care and indirectly correlated with the mediating effect of ethical sensitivity [38]. In a study conducted in Iran during COVID-19, a positive correlation was found between nurses’ ethical sensitivity and care behavior [4]. In a study conducted in South Korea, it was emphasized that the strongest determinant of person-centered care was ethical sensitivity [39]. Moral sensitivity is an important component that improves the quality of care for patients with COVID-19 [23–38].

According to the results of the present study, as nurses’ conflict subscale scores of moral sensitivity decrease, their compliance with isolation measures increases. An increase in nurses’ conflict scores indicates that they are less sensitive to internal conflict (less aware), whereas a low conflict score indicates that they are more sensitive to internal conflict (more aware). The more sensitive and aware nurses are of internal conflicts related to moral sensitivity, the higher their compliance with isolation measures. This may be related to the fact that new nurses do not have much experience with moral conflicts due to their lack of experience, as well as the fact that strict rules are applied in hospitals during the COVID-19 pandemic and that they are aware of the negative consequences that can be experienced regarding both their personal health and the health of patients if isolation measures are not followed. In the correlation and regression analysis between the conflict sub-dimension of moral sensitivity and compliance with isolation measures, although there was a statistically significant correlation, the value of the finding was low. This may be interpreted to mean that other factors, not examined in this study, also influence nurses’ compliance with isolation measures.

**Strengthens and Limitations**.

One of the strengths of the study is that the majority of the population resides in Istanbul, where most of the healthcare services are available. Cross-sectional data collection during the COVID-19 pandemic can be considered a limitation. Another limitation of the study is that the data were collected from nurses working in a single hospital. However, this hospital was preferred because it is a large hospital where complex patients can be treated, has clinics in all specialisms, and played a very active role in the COVID-19 pandemic. Finally, self-reported instruments were used in the study to assess nurses’ level of moral sensitivity and compliance with isolation precautions. Consequently, the findings are confined to individual reports.

## Conclusion

In this study, it was shown that when conflict sensitivity was high in nurses, their compliance with isolation measures was also high. To increase nurses’ compliance with isolation measures, it is important to reduce their experiences of internal conflict. The resolution of internal ethical conflicts requires individuals to review their ethical values, carefully evaluate the situation, consider the possible consequences, and follow an ethical decision-making process. Feedback from colleagues and managers on nurses’ behavior can also be helpful. When nurses start working in a hospital, it would be useful to start training on increasing their ethical sensitivity, particularly their sensitivity to conflict, during the orientation period. Providing training and counseling to increase the moral sensitivity of new nurses and all staff may contribute to patient care. Education programs and nursing administrators should provide a curriculum that emphasizes the development of various dimensions of moral sensitivity before graduation and provide continuous education to nurses after graduation. Case studies and case interpretations can be shared with staff to increase the participants’ level of moral sensitivity and compliance with isolation measures. To enhance the paper’s robustness, future studies could incorporate multiple hospitals, employ observational methods in addition to self-reports, and possibly look into more variables that might impact the relationship under study. Training only in compliance and ethics does not guarantee the adequacy and effectiveness of infection control and behaviors in realistic scenarios, so it may be useful to include sensitivity training and to examine other factors related to isolation precautions in the future. The relationship between team and organizational values, attitudes and behaviors (such as prejudices, cultural differences, problem-solving skills, critical thinking skills, relationships with colleagues, managers, or mentors, characteristics of role models, and work-family conflict) and compliance with isolation measures can be examined in future studies.

## Data Availability

The datasets (and raw data) used and/or analysed during the current study are available from the corresponding author upon reasonable request. Correspondence and requests for materials should be addressed to HTŞ.
